# Long Non-coding RNA in Neurons: New Players in Early Response to BDNF Stimulation

**DOI:** 10.3389/fnmol.2016.00015

**Published:** 2016-03-02

**Authors:** Vincenza Aliperti, Aldo Donizetti

**Affiliations:** Department of Biology, University of Naples Federico IINaples, Italy

**Keywords:** long non-coding RNA, BDNF, immediate-early genes, neuronal gene expression, brain diseases

## Abstract

Brain-derived neurotrophic factor (BDNF) is a neurotrophin family member that is highly expressed and widely distributed in the brain. BDNF is critical for neural survival and plasticity both during development and in adulthood, and dysfunction in its signaling may contribute to a number of neurodegenerative disorders. Deep understanding of the BDNF-activated molecular cascade may thus help to find new biomarkers and therapeutic targets. One interesting direction is related to the early phase of BDNF-dependent gene expression regulation, which is responsible for the activation of selective gene programs that lead to stable functional and structural remodeling of neurons. Immediate-early coding genes activated by BDNF are under investigation, but the involvement of the non-coding RNAs is largely unexplored, especially the long non-coding RNAs (lncRNAs). lncRNAs are emerging as key regulators that can orchestrate different aspects of nervous system development, homeostasis, and plasticity, making them attractive candidate markers and therapeutic targets for brain diseases. We used microarray technology to identify differentially expressed lncRNAs in the immediate response phase of BDNF stimulation in a neuronal cell model. Our observations on the putative functional role of lncRNAs provide clues to their involvement as master regulators of gene expression cascade triggered by BDNF.

## The Essential Role Of Bdnf In Neuron Life

Brain-Derived Neurotrophic Factor (BDNF) has been shown to have a central function in both neuronal development and in the adult nervous system. Knockout mice for BDNF usually die soon after birth and suffer developmental defects in the brain and sensory nervous system (Ernfors et al., 1995). Suppression of BDNF expression results in defective long-term potentiation (LTP) and memory formation (Korte et al., 1995; Linnarsson et al., 1997; Ma et al., 1998; Mu et al., 1999). In contrast, treatment of hippocampal slices from BDNF knockout mice with recombinant BDNF completely reversed deficits in LTP and significantly improved deficits in basal synaptic transmission (Patterson et al., 1996). BDNF interaction with NTRK2 receptor activates three signaling pathways: PI3K-Akt (PI3K, phosphatidylinositol-3 kinase), Ras-MAPK (MAPK, mitogen-activated protein kinase), and PLCγ-Ca^+^ (PLC, phospholipase C; Duman and Voleti, 2012). It is also known to regulate a large spectrum of processes of the nervous system, including cell survival, growth, and differentiation (Casaccia-Bonnefil et al., 1999; Bibel and Barde, 2000; Huang and Reichardt, 2003; Park and Poo, 2013; Suliman et al., 2013; Zagrebelsky and Korte, 2014), synaptic plasticity of neurons, and LTP (Xu et al., 2000; Bramham and Messaoudi, 2005; Gottmann et al., 2009; Minichiello, 2009; Mizui et al., 2014; Zagrebelsky and Korte, 2014; Leal et al., 2015). BDNF’s roles in neuronal processes during development and adulthood, support its potential role in the pathogenesis and treatment of both neurological and psychiatric disorders (Pruunsild et al., 2007; Nagahara and Tuszynski, 2011; Weissmiller and Wu, 2012). In fact, BDNF exerts potent pro-survival and functional effects in models of neurological diseases such as Parkinson’s (Howells et al., 2000; van der Kolk et al., 2015), Huntington’s (Zuccato et al., 2008; Jiang et al., 2013), and Alzheimer’s diseases (Holsinger et al., 2000; Michalski and Fahnestock, 2003; Peng et al., 2005; Faria et al., 2014), as well as depression and other psychiatric disorders (Karege et al., 2002; Aydemir et al., 2005; Gonul et al., 2005; Cunha et al., 2006).

## Bdnf: The Molecular Mechanism Of Action

Understanding the molecular mechanisms of BDNF’s functions may help to develop efficient therapeutic strategies for brain diseases. The molecular cascade triggered by BDNF has largely been investigated in the adult hippocampus, which retains a high degree of synaptic plasticity into adulthood. The effects of BDNF on LTP have been largely assigned to effects on modulation of receptor trafficking and local protein synthesis at the synapse by local activation of the translation machinery (Leal et al., 2014). BDNF acts at different levels to increase translation activity by altering the phosphorylation of proteins involved in the initiation step of protein synthesis, such as the guanine nucleotide exchange factor eIF2B (Takei et al., 2001) and eIF4E and 4EBP1 (Takei et al., 2004). In addition, BDNF can affect the elongation step, as shown in cultured cortical neurons, where BDNF stimulation was shown to increase the phosphorylation (activation) of eukaryotic elongation factor 1A (eEF1A; Inamura et al., 2005; Leal et al., 2014).

## Bdnf And *De Novo* Gene Expression

Long-term effects of BDNF on synaptogenesis, synaptic plasticity, and cell survival often depend on *de novo* gene expression. BDNF first affects the expression of genes (immediate-early genes, IEGs) that are regulated directly downstream of its signal transduction pathways and couple early signals to late expression of the downstream target responsible for persistent changes in neuronal phenotype. Some BDNF-induced IEGs involved in synaptic plasticity have been identified. Among them, activity-regulated cytoskeleton-associated protein (*ARC*), salt-inducible kinase 1 (*SIK1*), and transcription factor *NR4A1* (*Nur77*), have been shown to be up-regulated early by BDNF stimulation of neurons (Ying et al., 2002; Rao et al., 2006; Zheng et al., 2009; Finsterwald et al., 2013). Beyond the classical protein-coding IEGs, micro-RNAs (miRNAs) have also been singled out in the early response to BDNF stimulation. For example, miR-132 has been shown to be markedly up-regulated early after BDNF treatment of cortical neurons and involved in neuron morphogenesis likely triggering a rapid and persistent downregulation of protein levels (Vo et al., 2005). Both the proteins and miRNA classes of regulators therefore seem to participate in the early response to BDNF stimulation to induce the expression of downstream effectors involved in long-term synaptic changes and cell survival. Mechanisms regulating the initial molecular cascade are likely even more intricate when considering that a new class of non-coding RNA has recently been emerging as a key regulator of gene expression: the long non-coding RNA (lncRNA).

## Long Non-Coding Rna: A New Class Of Regulatory Rna

Long non-coding RNA are a heterogeneous class of numerous transcripts mainly produced by RNA polymerase II and defined as RNA molecules of more than 200 bases in length with no protein-coding capacity. Although the functional role of the vast majority of lncRNAs transcribed in a cell has been subject to much debate (Palazzo and Lee, 2015), the literature shows many examples of their effects on gene expression regulation. The mechanism of action is based on their ability to interact with other molecules, such as DNA, RNA, and proteins. In this way, they can act at different stages of gene expression and in processes ranging from chromatin remodeling to transcriptional, post-transcriptional, and translational regulation (Vance and Ponting, 2014). In addition, a recent analysis of Ruiz-Orera et al. (2014) on ribosome profiling experiments provided important evidence that lncRNAs associated with ribosomes may play an important role in *de novo* protein evolution by encoding short peptides. A large fraction of tissue-specific lncRNAs is expressed in the brain (Derrien et al., 2012), and many reviews have emphasized their role in neurodevelopment, brain function, and a wide range of neurodevelopmental, neurodegenerative and psychiatric diseases (for instance, see St Laurent and Wahlestedt, 2007; van de Vondervoort et al., 2013; Wu et al., 2013; Barry, 2014; Roberts et al., 2014; Tushir and Akbarian, 2014). An emblematic example is the antisense transcript, Bdnf-AS (Pruunsild et al., 2007). Recently, Modarresi et al. (2012) demonstrated that *Bdnf*-AS downregulates *Bdnf* expression through a role in the guidance, introduction, and maintenance of H3K27me3 involving PRC2-mediated repressive chromatin remodeling. Single-stranded oligonucleotides and siRNA-mediated depletion of Bdnf-AS in adult mouse brain resulted in a several-fold increase in Bdnf transcript and protein in the hippocampus and frontal cortex (Modarresi et al., 2012). These results paved the way for considering lncRNA as potential drug targets and to search for new strategies to inhibit *BDNF*-AS function to treat a number of neurological diseases in which *BDNF* is downregulated. Due to their growing relevance in the regulation of neuronal gene expression, we pondered whether lncRNAs may have a role in the regulatory cascade triggered by BDNF stimulation. In particular, we focused on immediate early response to the neurotrophin stimulation to gain insight into the potential involvement of lncRNAs as a master regulator in BDNF-induced neuronal transcriptional changes.

## Rationale, Materials And Methods

### Neuronal Cell Model and Culture Conditions

We used the popular SHSY-5Y cell line as a neuronal cell model for BDNF stimulation. SHSY-5Y is a neuroblastoma cell line that can be propagated by easy and low-cost methods when in its native undifferentiated status (Kovalevich and Langford, 2013). SHSY-5Y can be differentiated into cells with a more mature and neuron-like phenotype (Kovalevich and Langford, 2013). In particular, for our study, SH-SY5Y cells (ATCC^®^) were grown and propagated in Dulbecco’s modified Eagle’s medium (DMEM, Microtech^®^), supplemented with 2 mM L-glutamine (Lonza BioWhittaker^TM^), and a solution of 1% penicillin/streptomycin (Lonza BioWhittaker^TM^) and 15% Fetal Bovine Serum (FBS, Gibco^®^). The SHSY-5Y line comprises at least two morphologically and biochemically distinct phenotypes: neuroblastic (N-type) and a low proportion of epithelial-like (S-type; Encinas et al., 2000) phenotypes. For BDNF treatment on a more homogeneous neuronal cell population, we performed an enrichment procedure based on the different substrate adherence between the two cell phenotypes. The obtained N-enriched population of SHSY-5Y was differentiated by decreasing FBS concentration from 15 to 1.5% and adding 10 μM of RA (retinoic acid-RA, Sigma–Aldrich^®^) for 6 days (the medium was refreshed every 2 days). This produced differentiated cells responsive to BDNF, since RA treatment induces the expression of NTRK2 receptor in SH-SY5Y cells (Kaplan et al., 1993; Encinas et al., 2000). After 6 days of differentiation, the medium containing 1.5% FBS and RA was removed and substituted with a medium without FBS for two groups of cells. One of these groups was treated with 10 ng/mL of BDNF (PeproTech^®^) for a specific time, whereas the second group was not treated and used as a control for the gene expression analysis. We carried out a preliminary investigation to choose the most appropriate time for analyzing the immediate early response to BDNF treatment. We based this analysis on the expression pattern of three immediate early genes downstream of BDNF signaling activation: ARC, NR4A1, and SIK1. The mRNA level of all these genes peaked at 1 h of BDNF treatment (data not shown). Based on these results, microarray analysis was carried out on RNA extracted after 1 h BDNF-treatment.

### RNA Isolation and Quality

Total cellular RNA was isolated using an RNeasy^®^Mini Kit (Qiagen) according to the RNeasy^®^Mini Handbook (Qiagen). DNA contamination was efficiently removed by on-column DNAse digestion (Qiagen). The concentration and the purity of the RNA sample were assessed using NanoDrop^®^1000 (Thermo Scientific). Total RNA quality was assessed by an Agilent 2100 Bioanalyzer (Agilent Technologies).

### Microarray Experiment Analysis

Microarray experiments were performed on biological triplicate samples. Microarray hybridizations were performed by the Transcriptomics and Genomics core facility of the Department of Emergency and Organ Transplants (DETO) – Nephrology Unit – of the University of Bari ‘Aldo Moro’ Italy^1^. The labeled cRNA was produced using a Low Input Quick Amp Labeling (LIQA) kit (Agilent Technologies) and hybridized for 17 h at 65°C on an Agilent SurePrint G3 8 × 60K custom lncRNA expression array (Agilent Technologies). This array contains two probes for 22,001 lncRNAs targeting the Gencode v15 human lncRNA annotation, together with one probe for 17,535 randomly chosen protein-coding transcripts. After hybridization, the slide was washed according to Agilent protocols and scanned using a High-Resolution Microarray C Scanner (Agilent Technologies). The image file was processed using Agilent Feature Extraction software (v10.7.3). The microarray grid was correctly placed, and outlier pixels (which were rejected) and inlier pixels were identified. Normalization was performed according to the Quantile method. The differentially expressed probes were selected using a moderated *t*-test with a *p*-value cut-off of 0.05.

### Quantitative RT-PCR

qPCR validation was performed on independent biological replicates in triplicate. cDNA was synthesized from 1 μg of total RNA using an Invitrogen SuperScript III^®^kit. Real-time PCR was performed using the SYBR green method and an Applied Biosystems 7500 System. The PCR conditions included a denaturation step (95°C for 10 min) followed by 40 cycles of amplification and quantification (95°C for 35 s, 60°C for 1 min). Relative gene expression levels were normalized to the reference gene hypoxanthine phosphoribosyl transferase 1 (*HPRT1*) and calculated by the 2^-ΔΔCt^ method. The sequences of the primers used are reported in Supplementary Table S1.

### lncRNA Classification and Functional Analysis

The LNCipedia database^2^ (Volders et al., 2015) was used for retrieving the transcript ID, gene ID, and alternative gene name of the differentially expressed lncRNAs. Differentially expressed lncRNAs were classified by considering their position relative to adjacent protein-coding genes as reported by Mattick and Rinn (2015). Protein-coding potential of the differentially expressed lncRNAs was assessed by CPAT (Coding-Potential Assessment Tool) software^3^ (Wang et al., 2013). For the functional analysis of the differentially expressed lncRNAs, a list of nearby potentially regulated genes was retrieved using the computational tool GREAT (Genomic Regions Enrichment of Annotations Tool^4^; McLean et al., 2010). Functional enrichment analysis for the predicted target genes and differentially expressed coding genes was performed using the DAVID system (Database for Annotation, Visualization, and Integrated Discovery^5^), which uses Gene Ontology (GO) to identify the molecular function represented in the gene profile (Dennis et al., 2003). We obtained a list of potentially regulated miRNAs from lnCeDB^6^, a database that provides human lncRNAs (version Gencode 19) that can potentially act as competitive endogenous RNAs (ceRNAs) and interfere with the pathway of miRNAs (Das et al., 2014). These miRNAs were analyzed by miR2Disease^7^ to find miRNAs deregulated in human diseases (Jiang et al., 2009). The potentially regulated miRNAs were also analyzed by miRTarBase^8^ to find their experimentally validated mRNA targets (Chou et al., 2016). The list of the retrieved mRNA was analyzed by the DAVID tool. For all the DAVID analyses, the significance of enrichment of each GO term was assessed by a *p*-value of <0.05 and ranked by the number of differentially expressed genes (count).

## Results and Discussion

### Summary of Microarray Experiment Results

Long non-coding RNA expression profiles are summarized in **Table 1**. We found that 155 lncRNAs and 238 mRNAs were significantly differentially expressed (*p*-value <0.05). A fold change of >1.5 was found in 41 lncRNAs (24 up and 17 down regulated) and 40 mRNAs (31 up and nine down regulated; **Table 1**, Supplementary Table S2). A panel of both differentially expressed lncRNAs and mRNAs with fold change above 1.5 was validated by qPCR (Supplementary Figure S1). We analyzed the 41 differentially expressed lncRNAs by CPAT software to assess their protein-coding potential. We found that 37 lncRNAs have a coding probability value below the cutoff (0.364), while the remaining 3 lncRNAs have a coding probability value above the cutoff (Supplementary Table S3). Among these three transcripts we found C6orf176, the RNA with the highest fold change in our analysis (Supplementary Tables S2 and S3). The vast majority of the top regulated coding transcripts with fold change above 2 are classical immediate early genes involved in different biological processes in response to various neural stimuli, such as *NR4A3*, *ARC*, *EGR1*, and *DUSP5* (**Table 2**). A number of lncRNAs showed a fold change above 2, including the previously reported C6orf176 and MIAT (also known as Gomafu; **Table 2**). C6orf176 has been shown to be readily upregulated with a peak at the 2-h time point of treating human ocular ciliary smooth muscle cells with an EP2- and EP4-specific agonist (Reitmair et al., 2012). Notably, there was rapid and pronounced transcriptional upregulation followed by a brisk decline which resembles the kinetics of immediate early response genes (Reitmair et al., 2012). MIAT was recently shown to be acutely regulated in response to neuronal activation in mouse primary cortical neurons (Barry et al., 2014). In particular, this lncRNA is strongly downregulated after 1 h and 3 h of KCl depolarization (Barry et al., 2014). Interestingly, our data are in accordance with that of Barry et al. (2014), since we detected downregulation of MIAT transcript after 1 h of stimulation with BDNF, which is a well known activity-dependent factor.

**Table 1:** Summary of microarray analysis.

Transcript	Number	Differentially expressed	FC ≥ 1.5
lncRNA	22,001	155	41
mRNA	17,535	238	40


**Table 2:** Top regulated coding and long non-coding transcripts with fold change above 2.

Coding	Regulation	FC	lncRNA	Regulation	FC
NR4A3	Up	9.2	C6orf176	Up	3.8
ARC	Up	8.3	lnc-NPAS4-1	Up	3.1
RHOB	Up	4.3	lnc-WDR1-1	Up	2.8
fam46a	Up	3.6	IGFBP7-AS1	Up	2.1
EGR1	Up	3.0	lnc-ZSCAN10-4	Up	2.1
DUSP5	Up	3.0	MIAT-003	Down	2.1
KLF10	Up	2.3	MIAT-001	Down	2.0
MAP3K14	Up	2.0	lnc-RHOF-1	Down	2.0
F3	Up	2.0			


### Classification and Functional Analysis of lncRNA Regulated by BDNF

**FIGURE 1:**
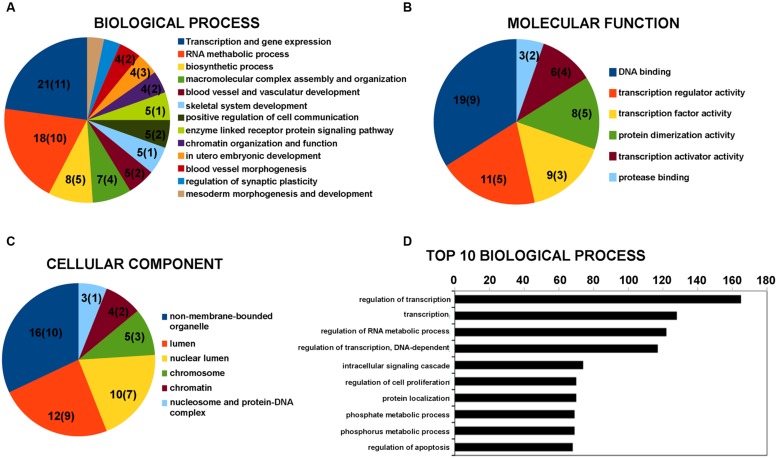
**Gene Ontology (GO) enrichment analysis for lncRNAs by DAVID bioinformatics tool.** **(A)** GO analysis of lncRNA-target genes + differentially expressed coding genes according to biological process. **(B)** GO analysis of lncRNA-target genes + differentially expressed coding genes according to molecular function. **(C)** GO analysis of lncRNA-target genes + differentially expressed coding genes according to cell component. **(D)** Top 10 biological processes for coding genes that are target of miRNA potentially regulated by the differentially expressed lncRNAs. Grouped GO terms are reported as following with the ID of the single GO term. Transcription and gene expression: GO:0045449 + GO:0006350 + GO:0006355 + GO:0006357 + GO:0045893 + GO:0045941 + GO:0010628 + GO:0045944; RNA metabolic process: GO:0051252 + GO:0051254 + GO:0045935 + GO:0051173 + GO:0010604; biosynthetic process: GO:0010557 + GO:0031328 + GO:0009891; macromolecular complex assembly and organization: GO:0065003 + GO:0043933 + GO:0034622 + GO:0034621; blood vessel and vasculature development: GO:0001568 + GO:0001944; chromatin organization and function: GO:0006334 + GO:0031497 + GO:0065004 + GO:0034728 + GO:0006323 + GO:0006333; regulation of synaptic plasticity: GO:0048168 + GO:0048167; mesoderm morphogenesis and development: GO:0048332 + GO:0007498. The number on the pie chart indicates the number of differentially expressed coding genes associated to the GO term (or GO group), while the number in brackets indicates the number of genes located near the differentially expressed lncRNAs.

The lncRNAs were classified in accordance with the definition discussed in “Materials and Methods” Section. The vast majority of differentially expressed lncRNAs were included in the intergenic and antisense classes (Supplementary Table S2). Five lncRNAs with a fold change above 1.5 were hypothesized to have a role in physiological and pathological processes in neuronal cells by regulating gene expression (**Table 3**). This supports the hypothesis of an involvement of lncRNA in BDNF-mediated molecular effects. To assess the potential function of the vast majority of the differentially expressed lncRNAs, we examined the function of genes located near the lncRNAs in the genome. The list of these coding genes and the list of differentially expressed coding RNAs found in our microarray survey were used for GO and pathway enrichment analysis with the DAVID tool. GO terms that shared the same genes were grouped together as shown in **Figure 1** with the ID of single GO terms. We report the number of genes associated with the GO group in a pie chart with the number of genes located near the lncRNAs in brackets. The most enriched biological processes are related to transcription regulation and RNA metabolic process (**Figure 1A**). In addition, there are also processes related to chromatin organization and function (**Figure 1A**). The involvement of genomic loci of lncRNA in transcription control was also revealed by GO terms of molecular function (**Figure 1B**). Regarding cellular component analysis, enriched GO are related to non-membrane-bound organelle, lumen and chromatin structure (**Figure 1C**).

**Table 3:** Differentially expressed lncRNA that have been identified in literature.

Gene symbol	Regulation	Function
MALAT1 (Neat2)	Down	Control of the expression of genes involved in synapse function (Bernard et al., 2010). Down-regulation led to cell arrest in the G1/S or G2/M phase (Yang et al., 2013).
MIAT (Gomafu)	Down	Down-regulated in response to neuronal activation and involved in schizophrenia-associated alternative splicing (Barry et al., 2014). Decreased in the medial prefrontal complex following fear conditioning and knockdown of promoted stress reactivity and anxiety-like behavior (Spadaro et al., 2015). Neurogenic commitment and neuronal survival, sustained overexpression of Miat promoted neuronal death (Aprea et al., 2013).
HAND2-AS1 (Dein)	Down	Highly expressed in stage IVS neuroblastoma (Voth et al., 2007). Expression is neuroblastoma is co-regulated together with HAND2 (Voth et al., 2009).
C6orf176	Up	A possible regulatory function in response to cAMP signaling (Reitmair et al., 2012).
HOXD-AS1	Up	It is induced by RA, could be regulated via PI3K/Akt pathway and controls genes involved in RA signaling, angiogenesis and inflammation (Yarmishyn et al., 2014).


### miRNA Targets on lncRNA

Long non-coding RNAs can affect gene expression by interfering with the microRNA pathways and acting as competing endogenous RNA. We therefore used the lnCeDB database to unravel microRNA (miRNA)–lncRNA putative functional interactions by identifying miRNAs with binding sites in differently expressed lncRNAs. Seven of the miRNAs found are associated with neuropathologies reported in the mir2disease database (**Table 4**). To acquire information on biological processes affected by the putative lncRNA-miRNA interaction, we retrieved a list of experimentally validated mRNA targets of all the previously identified miRNAs and carried out a functional analysis with the DAVID tool. In the top 10 biological processes, the most represented categories are related to the regulation of transcription and RNA metabolic processes (**Figure 1D**).

**Table 4:** Putative miRNA targets on lncRNA and their involvement in neuropsychiatric diseases.

miRNA symbol	Neuropathology
hsa-miR-339-5p	Neurodegeneration
hsa-miR-433	Parkinson’s disease
hsa-miR-133b	Parkinson’s disease
hsa-miR-346	Schizophrenia
hsa-miR-328	Alzheimer’s disease
hsa-miR-299-3p	Alzheimer’s disease
hsa-miR-422a	Multiple sclerosis


## Conclusion

The gene expression occurring immediately after neural stimuli plays critical roles in long-lasting neuronal changes. Further efforts are needed to isolate novel IEGs, with the hope of finding “master genes” for neuronal processes and subsequent therapeutic targets for brain diseases. The molecular mechanisms underlying the gene expression regulation in the initial phase of the stimulus-induced molecular cascade comprise the product of coding genes, miRNAs, and very recently, lncRNAs (Aitken et al., 2015). We sought to evaluate the potential role of lncRNAs in early gene expression regulation triggered by the neurotrophin BDNF. In particular, using microarray technology, we found that hundreds of lncRNAs and coding IEGs changed their transcript level after 1 h of BDNF treatment on neuronal cells. Some of the differentially expressed lncRNAs are already known. These are strictly associated with neuronal cellular processes and are involved in gene expression regulation. However, the biological functions of a vast majority of lncRNAs identified in this study are not currently understood. We therefore used computational approaches to provide preliminary insights into their potential functionality. The most enriched ontology terms were related to the transcription regulation processes, which highlights the putative role of lncRNAs in orchestrating the immediate response to BDNF. Overall, this study presents an interesting area for further investigations into lncRNAs with essential roles in molecular and cellular processes triggered by neurotrophins.

## Author Contributions

Conceived and designed the experiments: AD. Performed the experiments: VA and AD. Analyzed the data: VA and AD. Wrote the paper: AD.

## Conflict of Interest Statement

The authors declare that the research was conducted in the absence of any commercial or financial relationships that could be construed as a potential conflict of interest.

## Footnotes

1 http://www.uniba.it/ricerca/dipartimenti/deto

2 http://www.lncipedia.org/

3 http://lilab.research.bcm.edu/cpat/

4 http://bejerano.stanford.edu/great/public/html/

5 http://david.abcc.ncifcrf.gov/

6 http://gyanxet-beta.com/lncedb/

7 http://www.mir2disease.org/

8 http://mirtarbase.mbc.nctu.edu.tw/
