# Supplementary material for: Long Non-coding RNA in Neurons: New Players in Early Response to BDNF Stimulation
Source: Front Mol Neurosci. 2016 Mar 2;9:15. doi: 10.3389/fnmol.2016.00015 (PMC4773593; doi:10.3389/fnmol.2016.00015)
Supplement: Supplementary file 1 [file Table_1.DOC]

**Table S1. Sequence of forward (F) and reverse (R) primers used for qPCR validation.**

**Gene symbol Ensemble code Primer sequence**

NR4A3 ENSG00000119508 F_ 5’- TTTGGAGCTGTTTGTCCTCA -3’

R_ 5’- CACTCCCCAAATCCACGAAG -3’

ARC ENSG00000198576 F_ 5’- GAGTCCTCAAATCCGGCTGA -3’

R_ 5’- GCACAGCAGCAAAGACTTT -3’

EGR1 ENSG00000120738 F_ 5’- GAGCAGCCCTACGAGCAC -3'

R_ 5’- GAGTGGTTTGGCTGGGGTAA -3’

RHOB ENSG00000143878 F_ 5’- TTCGAGAACTATGTGGCCGA -3’

R_ 5’- GCACATGAGAATGACGTCGG -3’

ATF3 ENSG00000162772 F_ 5’- ATCACAAAAGCCGAGGTAGC -3’

R_ 5’- TCCTTCTTCTTGTTTCGGCAC -3’

SYBU ENSG00000147642 F_ 5’- AGAGCAGAGAGTTCAACCCC -3’

R_ 5’- AGCATCTGAGGGTGTCTTCA -3’

C6orf176 ENST00000455853 F_ 5’- GTCAGCATACTTTGGCGGAC -3’

R_ 5’- GTTGGTGCACGTGGGAGT -3’

lnc-NPAS4-1 ENST00000526186 F_ 5’- TAGACCACCTGAGGATGACC -3’

R_ 5’- AGAGGGCTGTCAAAGTGTGA -3’

RP11-182L21.2 ENST00000431300 F_ 5’- CACTTTGAGGTTCCCACTGC -3’ R_ 5’- TTGATGGCAGGGAGACGC -3’

HAND2-AS1 ENST00000505032 F_ 5’- TACGAAGACCTTGGGCGATT -3’ R_ 5’- GCGTTTAATGGTTCCCCTCC -3’

MIAT ENST00000423278 F_ 5’- CATGTGGTTCCAGACACGTT -3’ R_ 5’- CCTTCTGTCTCCTCTGTCCC -3’

LINC01089 ENST00000545885 F_ 5’- AGCAGAACGTGAGGGTGTAA -3’ R_ 5’- AGAGTCAAACTAGGCCTGCC -3’
