# Supplementary material for: Long Non-coding RNA in Neurons: New Players in Early Response to BDNF Stimulation
Source: Front Mol Neurosci. 2016 Mar 2;9:15. doi: 10.3389/fnmol.2016.00015 (PMC4773593; doi:10.3389/fnmol.2016.00015)
Supplement: Supplementary file 4 [file Image_1.PDF]

Long non-coding RNA in neurons: new players in early response to BDNF stimulation

Vincenza Aliperti[1], Aldo Donizetti[1*]

[*]Correspondence: aldo.donizetti@unina.it

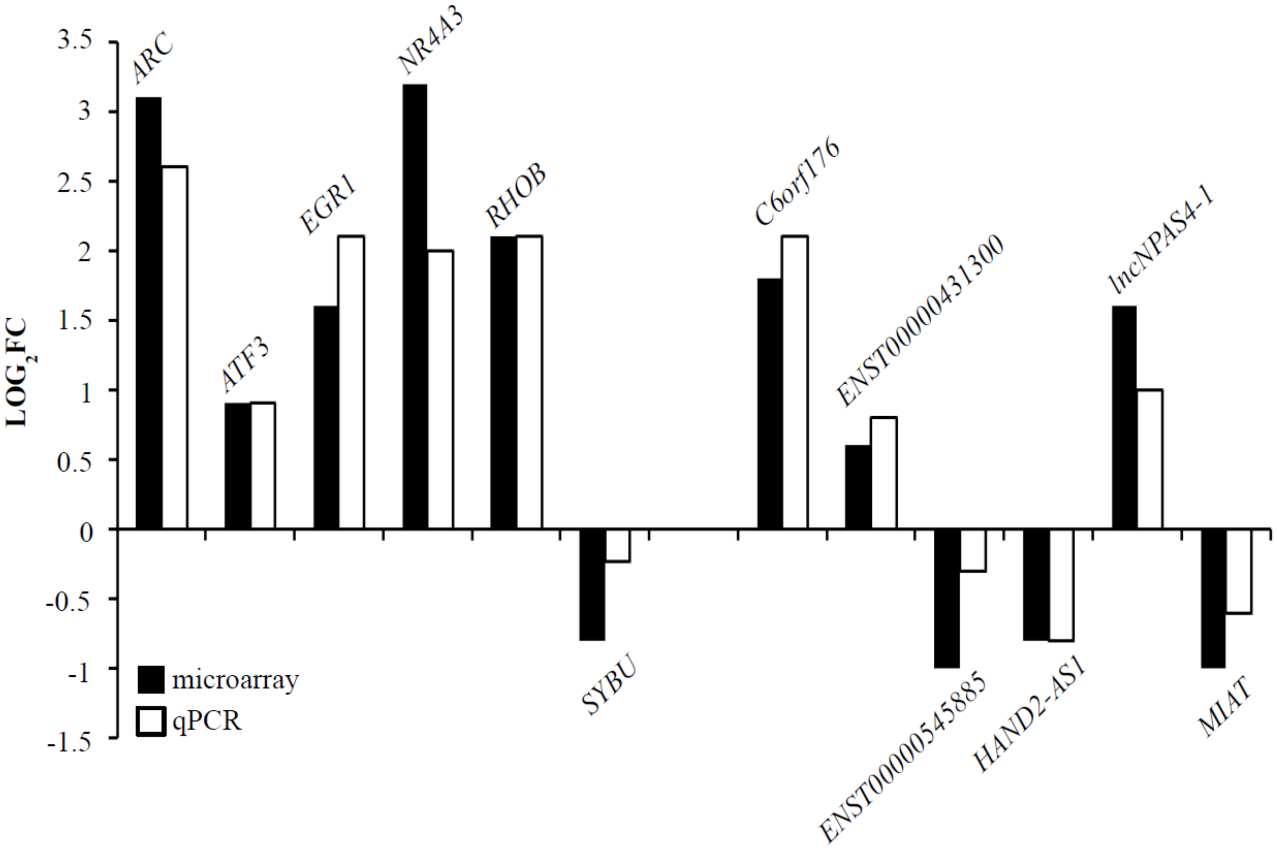

Supplementary Figure 1. qRT-PCR validation of some differentially expressed mRNA and lncRNAs.
